# Sublinear association between cortical thickness at the onset of the adult lifespan and age-related annual atrophy parallels spatial patterns of laminar organization in the adult cerebral cortex

**DOI:** 10.1016/j.ynirp.2021.100011

**Published:** 2021-05-17

**Authors:** Bruno Hebling Vieira, Carlos Ernesto Garrido Salmon

**Affiliations:** InBrain Lab, Universidade de São Paulo, Ribeirão Preto, Brazil

**Keywords:** Brain aging, Age-related atrophy, Cortical thickness, Cortical thinning, Morphometry

## Abstract

Brain aging is a complex process, entailing alterations at the most diverse levels of brain structure and functioning. On the macroscopical scale, gray matter atrophy is one of its most prominent markers, but it remains to be elucidated why some regions are more affected by it than others. In this work, we aimed to explore how age affects the morphometry of cortical structures, and specially the relationship between atrophy and the initial state of regions. To this end, anatomical T1-weighted images from 612 subjects aged 18–85 years were employed. Atlas-based cortical morphometric estimates were obtained. Our results show that, whilst there is no obvious shared pattern between the initial surface area of cortical regions at eighteen years and their yearly rate of decay, for cortical thickness there is enough evidence to suggest thicker regions at eighteen years of age also present higher yearly rates of thinning, although yearly percentages are proportionately smaller. Our analyses reveal that neocortical regions tend to conform to this trend, where agranular cortices experience higher atrophy levels than granular cortices, contrasting a few non-pure neocortical regions that do not follow this trend, highlighting parallels between brain evolution and brain aging.

## Highlights

• Inhomogeneous cortical atrophy is a marker of normal aging in humans.
• Yearly atrophy at the onset of adult life is related to the initial thickness.
• Thicker regions display higher rates of atrophy in millimeters per year.
• However, thinner regions exhibit greater percentage loss per year.
• This association is partially aligned with laminar organization patterns.

## 1 Introduction

High resolution neuroanatomical techniques based on magnetic resonance imaging facilitate the study of the brain morphology and how it changes due to age. Usual quantitative descriptors of cortical gray matter geometry include thickness and surface area, which have been shown to be genetically distinct (Winkler et al., 2010). In fact, there is evidence that ontogenetically driven age-related changes in these may reflect different aspects of neurobiological aging (Lemaitre et al., 2012).

Studies showed cortical thickness, surface area, volume and gyrification are reduced in the whole cortex due to age to varying degrees (Hogstrom et al., 2013; Fjell et al., 2009a, 2014a). While almost the whole cortex displays evidence of age-related thinning, frontal gyri, superior and middle temporal gyri, the temporoparietal junctions and pre- and postcentral gyri display highly consistent thinning, whereas inferior temporal lobes and anterior cingulate cortices are less affected (Fjell et al., 2009b). This thinning process starts at early age (Amlien et al., 2016), as the cortex attains peak thickness in most regions prior to or during puberty (Shaw et al., 2008), and affects differentially the opposing brain hemispheres (Zhou et al., 2013). In a study with 17075 subjects aged 3–90 years, Frangou et al. (2021) determined that age accounts for 59% of variance in cortical thickness.

Since the number of neuronal cells remains relatively constant throughout the lifespan of healthy adults (Freeman et al., 2008), age-related cortical atrophy is not simply attributable to loss of neuronal cells. Nor can atrophy be completely explained by the incidence of incipient or pre-symptomatic Alzheimer's Disease (Fjell et al., 2009c). The occurrence of changes in synapses and spines and shrinking of cell bodies are hypothesized to be possible key factors in these phenomena (Fjell et al., 2015). In the case of spines, a major decrease in spines has been observed, regardless of the distance to the neuron body (Benavides-Piccione et al., 2013).

In comparison to cortical thickness, that often presents strong atrophic trends in literature, cortical surface area tends to remain relatively constant throughout the adult lifespan. Global surface area remains relatively constant throughout adult life after 10–15 years of age, displaying only a mild brain-wide rate of atrophy (Raznahan et al., 2011; Lemaitre et al., 2012; Amlien et al., 2016). Cortical surface area expansion during early development might be a mechanism for cortical specialization, through the disentanglement of cortical connections (Seldon, 2005).

However, an often overlooked factor on cortical anatomy during aging is cytoarchitectonics, as most studies tend to focus on anatomical or functional subdivisions of the brain. Cytoarchitectonics are directly related to functional specialization and cortical communication. Cell-specific gene expression correlates of cortical thinning have been found (Vidal-Piñeiro et al., 2020), suggesting a cellular moderator of age-related trajectories. Valk et al. (2020) found genetic and evolutionary correlates of macroscale organization in cortical thickness, including two axes of organization: an inferior-superior axis and an anterior-posterior axis. The former aligns with the dual origin theory of cortical development while the latter aligns with a direction of functional topography, from sensory and perceptual areas to predominantly cognitive areas in the brain.

The cortex architecture is not uniform, its regional composition varies on an axis of the proportion of two broad types of cortex: allocortex and neocortex, sometimes called isocortex (Triarhou, 2007). The neocortex is evolutionarily younger and it is present on the outermost surface of the telencephalon (Zilles et al., 2004), occupying 91% of the cortical surface area. It houses higher-order cognitive functionalities, which are often impaired due to neurodegenerative disorders and other biological processes. On the other hand, allocortex is present in other gray matter structures such as the hippocampi (archicortex) and the rhinencephalic cortical portions, the piriform cortex (paleocortex) (von Economo, 2009). A striking difference between neocortex and allocortex is the number of cortical layers in their structures. Pure neocortex contains six neuronal layers from early stages of fetal development, whereas pure allocortex has between three and five layers (Zilles et al., 2004). Due to how myelin sheets originate during development, allocortex is also less myelinated than neocortex (Braak et al., 2003). Mesocortex, a transitional type of cortex, shares cytoarchitectonic and histological features of both neocortex and allocortex, being present in the insulas, and entorhinal, cingulate and parahippocampal cortices.

The neocortex is also heterogeneous, with regional variations in cytoarchitecture. Different areas have different laminar configurations, and this is readily perceived in the distribution of laminar thickness (von Economo, 2009). On opposite ends of a continuous spectrum we have cortices called granular and agranular (Zilles et al., 2004; von Economo, 2009; Solari and Stoner, 2011). They are named so due to the proportion of granule cells, i.e. small-body neurons, populations in their layers. Granular cortices are thinner and tend to dominate “input” areas such as the primary visual and primary sensorimotor cortices, while agranular cortices are thicker, with higher populations of large neurons, and predominate in “output” areas, such as the motor cortices (Solari and Stoner, 2011). Both agranular and granular cortices are classified as heterotypic, which means that they do not preserve an easily discernible six-layered architecture of other isocortical areas, accordingly called homotypic or dysgranular (Triarhou, 2007). Transitions between these types of cortex are gradual, and mixed regions are occur at the borders. For a topographical view of these cortical types, see Fukutomi et al. (2018) (https://www.sciencedirect.com/science/article/pii/S1053811918301058#Fig. 5).

Aging has been linked to cognitive decline and age-related atrophy cooccurs with changes in functional activity and connectivity (Vieira et al., 2020) and structural connectivity (Pinto et al., 2020). Understanding how age-related morphometric alterations affect the brain helps to unveil possible factors accounting for neuronal vulnerabilities, and how and why brain aging occurs. Even though we understand the lifespan trajectories of cortical gray matter morphometry, properties pertaining to such trajectories have not been studied in depth. For example, since it was found that cellular stress and ageing differentially affect allocortical and neocortical tissues (Posimo et al., 2013), morphometric trajectories might reflect that processes. In this work, we hypothesize spatial patterns of cerebral gray matter age-related atrophy are related to known structural properties of the brain, notably neocortical granularity gradients, which in turn are related to cortical thickness estimates. Absolute and relative rates of atrophy are not homogeneous in the cortex. We hypothesize that both these rates are related to the initial properties of the tissue. To test such hypothesis we employ a non-linear model relating yearly rates of atrophy with estimates of the initial state of tissue in a large population.

## 2 Methods

The NKI-RS Phase I and Phase II received express authorization by their Institutional Review Boards at the Nathan Kline Institute (#226781 and #239708) and at the Montclair State University (#000983 A and #000983 B). All participants provided written informed consent. See Nooner et al. (2012).

### 2.1 Demographics and imaging

Phenotypic data from 1148 subjects free of neurodegenerative disease symptoms were retrospectively obtained from the public databases maintained by the Nathan Kline Institute at Orangeburg, NY, that make up the Rockland Sample (NKI-RS) (Nooner et al., 2012), in their Pilot and Enhanced distributions, currently contributing 207 and 941 subjects each respectively, which are distributed through the International Neuroimaging Data-Sharing Initiative (INDI) and the 1000 Functional Connectomes Project (FCP) (Mennes et al., 2013). The NKI-RS is an ongoing large-scale endeavor aimed at phenotyping and imaging neurotypical subjects from the Rockland County, NY.

Exclusion criteria were age less than 18 years, left-handedness, and perceptible artifacts or defects in the anatomical scan detected by visual inspection. Applying these criteria, a final set of 613 subjects was achieved, 483 from the Enhanced NKI-RS and 130 from the Pilot NKI-RS subsamples.

The characteristics of the sample are shown in Table 1.

**Table 1:** Aging and gender characteristics of the sample. The asterisk denotes a significant difference to the age of females in the Enhanced NKI-RS.

Age (years)	Enhanced NKI-RS		Pilot NKI-RS	
	Male	Female	Male	Female
[18,27]	53	56	26	19
[28,37]	19	25	9	9
[38,47]	18	54	26	6
[48,57]	13	79	6	5
[58,67]	26	61	6	6
[68,77]	19	41	5	5
[78,87]	7	11	2	0
Mean	44.58	50.45	40.58	40.18
C.I.95%	(41.72, 47.44)	(48.49, 52.42)	(36.60, 44.55)	(35.15, 45.21)
Total	155	327	80	50

Anatomical scans comprised of high definition 3D MP-RAGE (Mugler and Brookeman, 1990). The image acquisition parameters are shown in Table 2.

**Table 2:** Image acquisition parameters in 3 T scanners. TR is the repetition time, the time between each volumetric acquisition. TE is the echo time, the time between consecutive echoes in the MP-RAGE acquisition. TI is the inversion time; it is the time between the inversion of the magnetization and the excitation pulse. FOV is the field of view in the antero-posterior, right-left, and foot-head directions, respectively. PA means parallel acquisition acceleration.

	Enhanced NKI-RS	Pilot NKI-RS
Voxel size (mm^3^)	1 × 1 × 1	1 × 1 × 1
TR (ms)	1900	2500
TE (ms)	2.52	3.5
TI (ms)	900	1200
Flip angle	9°	8°
FOV (mm^3^)	250 × 250 × 176	256 × 256 × 200
PA factor	2	–
Acquisition mode	Single-shot ascending	Single-shot ascending
Measurements	1	1
Acquisition time	4′18″	10′42″

### 2.2 Pre-processing

This pre-processing pipeline has been reported elsewhere (Vieira et al., 2020; Vieira and Salmon, 2019) and is partially reproduced below.

All anatomical images were pre-processed in Freesurfer v.6.0.0 (Fischl, 2012), based on the default “recon-all” routine. We obtained the segmentation of brain tissue types, and also the automatic whole brain parcellation (Fischl et al., 2002) of cortical gray matter.

Sulci and gyri present consistent differences in morphometry and structure: for example, sulci are consistently thinner than neighboring gyri (von Economo, 2009). For this reason we opted to use the Destrieux atlas (Destrieux et al., 2010) to perform our analyses, as it parcellates the cortex into 148 anatomical gyral-sulcal regions of interest (ROIs).

We qualitatively examined processing time and also cortical thickness estimates to identify artefactual measurements. A single subject was removed from the study due to faulty structural pre-processing, identified through long processing time.

We used GNU Parallel (Tange, 2015) to perform pre-processing in parallel, greatly reducing total computation time. Processes were carried through the resource management tool SLURM (Jette et al., 2002) in a single machine.

Regional cortical surface area and thickness estimates of the cortical structures defined in the Destrieux atlas were obtained from surface representations for each subject.

We opted to use Freesurfer v.6.0.0, since it is the most popular toolbox for cortical thickness estimation in the literature. It is known from the literature that different software choices leads to systematic differences in estimated cortical thickness (Kharabian Masouleh et al., 2020), even considering different versions of the same software (Vieira and Salmon, 2017). For example, CAT12 is known to deliver systematically higher estimates than Freesurfer (Kharabian Masouleh et al., 2020; Seiger et al., 2018), while using different versions of Freesurfer does not appear to induce difference in estimated age-related cortical thinning (Vieira and Salmon, 2017). For this reason, it is not guaranteed that a similar analysis performed with another toolbox, *i.e.* CIVET or CAT12, would lead to the same results. This points to the necessity of further studies exploring these phenomena using different data and software. Having said that, differences between toolboxes are submilimetric, there is substantial agreement, and deviations are age-independent (Seiger et al., 2018).

### 2.3 Statistical analysis

Given morphometric measurements $M=\left(M_{ij}\right)\in R^{n\times m}$, for *n* subjects and *m* regions, these can be modeled as linear functions of the subjects age. We model all interactions with subjects sex and also the dataset they came from, as presented in Table 1, to accommodate possible bias in the measurements due to the small difference in the parameters shown in Table 2. This is represented in Equation (1), where $Sex_{i}\in \left\{-1,1\right\}$, $Set_{i}\in \left\{-1,1\right\}$. This model allows different intercepts and slopes per each combination of sex and age.

(1) $$M_{i,j}=\beta_{0,j}+\beta_{Age,j}\cdot Age_{i}+\beta_{Sex,j}\cdot Sex_{i}+\beta_{Set,j}\cdot Set_{i}+$$

$$\beta_{Age:Sex,j}\cdot Age_{i}\cdot Sex_{i}+\beta_{Age:Set,j}\cdot Age_{i}\cdot Set_{i}+$$

$$\beta_{Set:Sex,j}\cdot Set_{i}\cdot Sex_{i}+\beta_{Age:Sex:Set,j}\cdot Age_{i}\cdot Set_{i}\cdot Sex_{i}+\varepsilon_{i,j}$$

We arrange the unbiased estimates of $\beta_{0,j}$ and $\beta_{Age,j}$, for each structure defined in the atlas, as the elements of the column matrices $\beta_{0}$ and $\beta_{\mathrm{Age}}$. We then define the expected initial value of the morphometric estimates, $I_{M}=\beta_{0}+18\cdot \beta_{\mathrm{Age}}$ and the percentage rate of yearly change at this age, $D_{M}=100\cdot \text{diag}{\left(I_{M}\right)}^{-1}\cdot \beta_{\mathrm{Age}}$. We studied this ratio because some cortices are thicker than others. For example, the same rate of yearly change in thickness in cortices with differing initial cortical thickness affects them unequally: the thinner cortex loses more thickness per year, proportionally. This procedure keeps all regions in a similar scale. The model of interest is shown in Equation (2).

(2) $$D_{M}=\gamma_{0}+\gamma_{1}\cdot I_{M}+\varepsilon$$

To quantify these associations, for both cortical thickness and regional surface area we fitted robust linear models (Maechler et al., 2016). Due to the relationship induced by $I_{M}$ in the ratio $D_{M}$, spurious correlations are induced. We follow Kronmal (1993) and estimate the model described in Equation (3) instead. This model is the expansion of Equation (2). The heteroskedasticity induced by the product in the residual term is accounted by weighting observations by $I_{M}^{-1}$.

(3) $$\beta_{\mathrm{Age}}=\alpha_{0}+\alpha_{1}\cdot I_{M}+\alpha_{2}\cdot I_{M}^{2}+\text{diag}\left(I_{M}\right)\varepsilon$$

Statistical analyses were conducted in the GNU distribution of the statistical language and environment R version 3.5.0 (R Core Team, 2019). Statistical maps on the *fsaverage* cortical surface were generated using custom code based on the “freesurfer_statsurf_display” MATLAB toolbox (Developmental Imaging Group - MCRI, 2017). Multiple comparisons were adjusted at the sub-analysis level, meaning that each result presented has been individually adjusted, using the Benjamini-Hochberg False Discovery Rate (FDR) controlling procedure, as it is also valid under positive dependency (Benjamini et al., 1906).

Motivated by anonymous reviewers, additional analyses were conducted taking into account gyral-sulcal differences. We added independent coefficients for sulcal and gyral regions. Fissurae were grouped with sulcal regions while poles were grouped with gyral regions. 18 regions are labelled as containing both gyri and sulci in the Destrieux parcellation. That is because some gyri and sulci are either inconsistent, too small or could not be easily delineated in the cortical surface (Destrieux et al., 2010) In a first analysis we opted to model these regions as gyral. In a second analysis we modeled these regions as being both sulcal and gyral, *i.e.* it shares the coefficients of both gyri and sulci.

Another interesting neurobiological question regarding granularity arose during peer review. To test whether granularity is associated with cortical thinning, we defined two groups of regions. The first group (n = 16, counting bilateral regions) contains less granular regions. It consists of the superior, middle, inferior pars triangularis frontal gyri, the superior frontal sulci, the short insular gyri, the anterior, middle-anterior and middle-posterior cingulate gyri and sulci. The second contains more granular regions (n = 8). It consists of central and calcarine sulci, occipital poles and anterior transverse temporal gyri (of Heschl). This selection was based on von Economo (von Economo, 2009) and Shamir et al. (2019), and can be seen in Supplementary Figure S1. We performed the Mann–Whitney *U* test to compare both groups regarding absolute and percentual yearly thinning.

## 3 Results

In total, 133 ROIs showed a significant effect of age on cortical thickness at the 0.05 FDR level. These ROIs are equivalent to 89.9 percent of the cortical structures studied, 88.7 percent of the cortical volume, and 88.9 percent of the cortical surface area. All of them had thinning cortices with aging on the average of the groups studied. For more details, see Supplementary Figure S2. For this analysis, the regional proportion of explained variance is shown in Supplementary Figure S3.

In total, 101 ROIs showed a significant effect of age on cortical surface area at the 0.05 FDR level. These ROIs are equivalent to 68.2 percent of the cortical structures studied, 76.8 percent of the cortical volume, and 75.1 percent of the cortical surface area. Of these, only three small ROIs had increasing area with age. See Supplementary Figure S4.

**Fig. 1:**
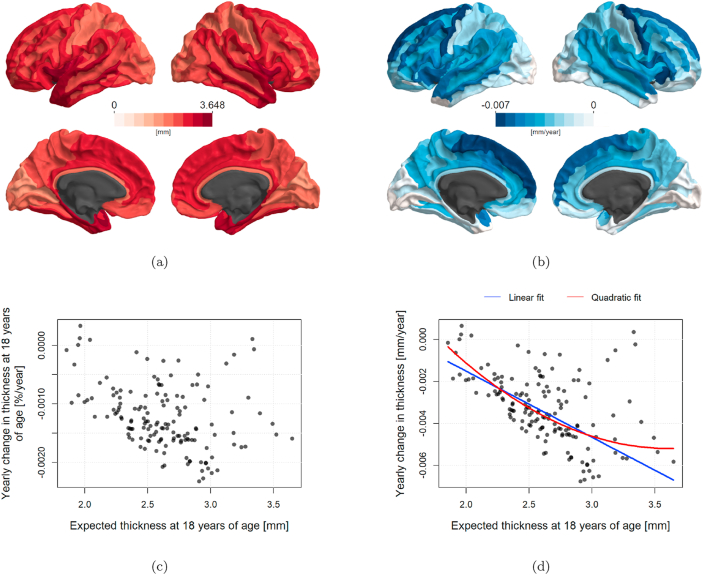
Dependence between yearly change and initial thickness of 148 cortical regions defined in the Destrieux atlas. (a) The expected cortical thickness obtained from Equation (1). (b) The expected absolute yearly cortical thickness atrophy according to Equation (1). (c) Yearly percentage change in thickness versus the expected thickness at the age of 18 years. (d) Yearly thickness change versus the expected thickness at the age of 18 years. The red continuous line represents a robust linear model like in Equation (3). The blue line represents a similar model without the quadratic term.

In Fig. 1(c) it can be noted that the yearly rate of cortical atrophy, shown in Fig. 1(b), increases with the initial thickness of the cortex, shown in Fig. 1(a). A robust linear regression model was fitted as described in Equation (3). The linear term $\alpha_{1}=-0.01138$, [(mm/year)/mm] is significant ($p_{\text{ANOVA}}<0.001$) The inclusion of the quadratic term $\alpha_{2}=0.00158$, [(mm/year)/mm^2^] is also significant ($p_{\text{ANOVA}}=0.001184$), resulting in a 29.27% increase of multiple-$R^{2}$, from 0.226 to 0.292. This means that while thicker cortices present higher yearly rates of atrophy, on average thinner cortices undergo more severe atrophy, proportionately. This effect can be visualized as the tapering of the curvature near the right end of the graph. The regions in the top right corner of Fig. 1(d) illustrate the need to prevent large deviations from dominating the model through the use of the robust linear model. For modeling taking into account gyral-sulcal differences, see Supplementary Figure S5.

$\alpha_{2}$ quantifies the dependence between the percentage yearly atrophy and the initial expected thickness. A positive $\alpha_{2}$ signifies that thicker cortices exhibit less atrophy than thinner cortices. $\alpha_{1}$ on the other hand is the coefficient that measures the direct proportionality of yearly atrophy and the expected value of thickness at 18 years of age. The negative value found was expected, meaning that thicker cortices lose, in absolute, more thickness than thinner ones. Thus, Fig. 1 shows that yearly percentage atrophy of cortical thickness depends on the baseline thickness of a region in a sublinear fashion, i.e. thicker regions lose more millimeters per year, though proportionately less than thinner regions.

**Fig. 2:**
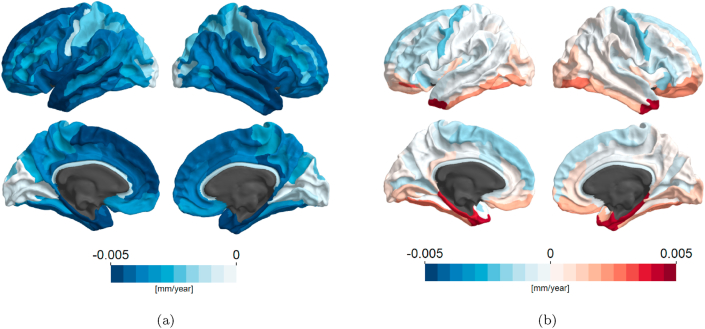
Expected yearly rate of atrophy (a) and residuals (b) according to the model in Equation (3).

The fitted values and the residuals of Fgiure 1(d) are shown in Fig. 2. The main apparent effect captured by the model is encoded as the expected values in Fig. 2(a). Secondary effects, which a perceptive deviations from the main tendency, are encoded as the residuals in Fig. 2(b). Residuals are low on most of the cortex, with the exception of both temporal poles and both parahippocampal gyri, where the observed atrophy is much lower than the expected. Note that the robust linear model employed admits non-zero correlation between residuals and expected values. In the case of Fig. 2, however, this correlation is not significant ($\rho =-0.123,\;\text{C}.\text{I}._{95\text{\%}}=\left[-0.279,0.0392\right]$)

**Fig. 3:**
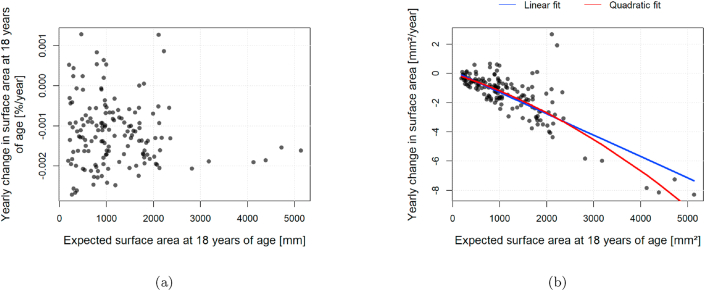
Dependence between yearly change and initial surface area of 148 cortical regions defined in the Destrieux atlas. (a) Yearly percentage change in surface area versus the expected surface area at the age of 18 years. (b) Yearly surface area change versus the expected surface area at the age of 18 years. The red continuous line represents a robust linear model like in Equation (3). The blue line represents a similar model without the quadratic term.

Analogously, the same results are shown for cortical surface area, in Fig. 3. The representations on the cortical surface has been omitted because no clear patterns of dependence were observed. A minute, albeit significant, quadratic effect analogous to the one in Fig. 1 is also observed ($p_{\text{ANOVA}}=0.001253$). Here, however, the inclusion of the quadratic term from Equation (3) increases multiple-$R^{2}$ by only 3.64%, from 0.624 to 0.646.

As shown in Equation (1), sex and dataset where also present in the morphometric linear models. The expected value of thickness at 18 years old is significantly different between sexes in 12 regions of the Destrieux atlas and also different between the two subsamples in 72 ROIs. No significant difference in the age-related thickness rate of change was observed between sexes. The effect of age on cortical thickness differed significantly between the subsamples in a single region, the right Inferior segment of the circular sulcus of the insula. For cortical surface area the same significant effects were observed, with 114 regions showing differences between sexes, 50 regions showing differences between subsamples, 2 regions showing differences in the effect of age between sexes, and no regions displaying different effects of age between subsamples. The triple interaction term in Equation (1) was not significant in any region for both cortical thickness or cortical surface area.

For both yearly absolute decay, in mm/year, and percentual decay, in %/year, the Mann–Whitney *U* test resulted in significant differences between higher and lower granularity regions (p≪0.001).

## 4 Discussion and conclusions

In all analyses, age was entered as an independent variable. In both NKI-RS sub-samples, age is recorded as a positive integer. This small censoring due to rounding was not accounted for in the models and should not alter the results.

Our choice of including subjects aged 18 years or more is justified by the literature. Shaw et al. (2008) shows that cortical regions peak no later than approximately 13 years of age. Although several regions peaked earlier than that, we were interested in analyzing the trajectories of the adult lifespan, hence our choice.

Our morphometric results support the notion of generalized age-related atrophy in the brain, with diminishing dimensions in gray matter structures. These findings have been widely reported in the literature (Fjell et al., 2009a, 2009b, 2009c, 2010, 2014a, 2014b, 2015; Walhovd et al., 2011; Lemaitre et al., 2012; Zhou et al., 2013; Harada et al., 2013; Lockhart and DeCarli, 2014; Storsve et al., 2014; Frangou et al., 2021). While our primary aim was not to characterize these trajectories, the convergence of results reassures us that we are capturing the actual age-related effects.

Particularly, as seen in Figure S2, thinning attains higher yearly rates in the Precentral gyri and the Superior frontal gyri, in accordance with the literature (Fjell et al., 2009b). Spared areas include several bilateral Occipital gyri and sulci with some Temporal gyri also being present, more specifically the temporal poles and parahippocampal gyri. As seen in Figure S3, inter-individual variability manifesting itself as low $R^{2}$ in our analyses appears in frontal-rostral and temporal regions, such as described in Frangou et al. (2021). However, in addition to these regions, occipital and postcentral gyrii also display higher levels of unexplained variance.

Cortical surface area reductions are prominent in Subparietal sulci, Lateral orbital sulci and several Temporal and Frontal gyri and sulci. Area is significantly increased with age in the Central sulci.

There is no exact spatial correspondence between age-related changes in cortical surface area and cortical thickness (Hogstrom et al., 2013). The phylogenetical principle of area maximization in lieu of cortical thickening for better functional organization (Seldon, 2005) could explain this phenomenon.

Even though aging is major factor for intersubject variability in morphometric estimates it explains on average 16% (14%–18% C.I.) of the variance in cortical thickness, 21% (20%–22% C.I.) of the variance in volume and 15% (15%–16% C.I.) of the variance in surface area among all cortical regions in our results. That does not necessarily mean a more complex model would be useful, since it makes sense for intersubject variability to exist due to other factors, several of which are not, and perhaps could not be, taken into account in our study. The linear hypothesis is a reasonable choice to the study of cortical thinning across the adult lifespan (Fjell et al., 2009b; Raznahan et al., 2011).

The analysis of initial and age-related percentage change of surface area of anatomical regions in Fig. 3 did not reveal any evident structure. Regional cortical surface area is relatively stable to aging, with smaller regional variations in the rates of decay than cortical thickness (Lemaitre et al., 2012). While in the cortex thickness diminishes due to changes in its own architecture, regional changes in area can be attributed to global volumetric changes. It then makes sense that areal changes are independent of the initial regional surface area.

To the best of our knowledge, for the first time we have quantitaively demonstrated that the rate of thinning is proportional to the initial thickness of the region, in Fig. 1. That means that thicker regions display higher rates of thinning than thinner regions, although they lose, proportionately, less thickness percentage per year compared with thinner regions.

In the neocortex, thicker cortices are agranular while thinner cortices are granular. We can hypothesize that age-related cortical thinning affects more regions with higher proportions of small-body neurons, i.e. granule cells. These regions, albeit thinner, have higher cell densities than agranular cortices (Triarhou, 2007).

The expected value of thickness can be thought of as a stand in for the granularity in the case of the neocortex, as agranular cortices are thicker whereas granular cortices are thinner. Previous results from the literature suggested the developmental trajectories of different cortices closely resemble their granularity (Shaw et al., 2008) Agranular cortices, being phylogenetically younger and performing higher cognitive functions, have more complex trajectories, closely matching the association cortices, which are expanded in humans in comparison to other mammals (Croxson et al., 2017). Our results demonstrate these cortices are also likely to show higher rates of atrophy due to aging, although losing proportionately less thickness than thinner cortices. This implies that higher cell density or, alternatively, higher proportion of granule cells, implies higher percentage loss of cortical thickness. The significant difference between absolute and percentual yearly cortical thickness atrophy in more and less granular regions adds supporting evidence to our hypothesis that age-related thinning is associated with granularity.

This result matches the hypothesis known in the literature as “last-in first-out”, where regions maturing, in evolution and development, last are the most likely to be affected by age-related declines (Raz, 2000). Indeed, decreases in the populations of large cell-body neurons during aging have been reported elsewhere (Martínez-Pinilla et al., 2016).

The observed sparing of the temporal poles is elusive though, as it is among the thickest cortices in the brain. It is a mixed cortex in the temporal lobe, containing granular, dysgranular, and agranular cortical sub-fields (Kondo et al., 2003; Pascual et al., 2015) and even agranular periallocortical elements (Zilles et al., 2004). Albeit six-layered, it can be considered agranular, since layers II and IV are sparsely populated with cells towards the pole (Triarhou, 2007).

In Fig. 2(a), we see how cortices are positioned in a gradient regarding their alignment to the association between atrophy and initial thickness. We argue this distribution closely resembles neocortical granularity, with more agranular cortices being blue and granular cortices white. See Triarhou (2007) for the spatial distribution of such cortices in the brain. Consistently, the anterior insulas contain agranular cortices, and are markedly positive. This suggests agranular cortices have higher rates of atrophy as well. In general, gyri are ranked higher than neighboring sulci in this direction. This corresponds to the fact that sulci are in general thinner than gyri (von Economo, 2009), and therefore show lesser effects of atrophy. See Figure S2.

Myelination, a process that happens through the lifespan, may also play a role in this. Myelination starts early in life in the primary fields and increases until reaching the mesocortical regions (Braak et al., 2003). Notably, the medial occipital lobe, the pericallosal sulcus and the central sulcus present higher iron concentration and myelination than the rest of the brain in all cortical depths (Marques et al., 2017; Rowley et al., 2015). These regions are the thinnest and present the lowest yearly rates of atrophy in the brain. The medial frontal lobe and the temporal gyri are among the thickest regions in the brain and likewise present smaller cortical myelin content.

In Fig. 2(b) we see a gradient orthogonal to the main effect, or in other words the deviations from the preferential direction. Since most superior cortices, mostly neocortical, have low scores in this scale and that the insular gyri, temporal poles and parahippocampal gyri, which are known to contain mesocortex, have high scores, which means that while they are thicker they present smaller rates of atrophy, we tentatively identify in this direction as a correlate for cortical type admixture, in a way that neocortical regions are generally ranked lower than allocortex-containing regions. Supplementary Figure S6, reproduced from Valk et al. [18, Figure 4], provides a visualization of the location and distance to focii of allocortex in the brain. This identification is not without issues though, since mesocortical and allocortical regions are much smaller than isocortical areas in humans, and therefore regions demarked from anatomical landmarks, such as the gyral-sulcal atlas employed, tend to cover several cytoarchitectural distinct areas. The pericallosal sulcus, a region containing periarchicortex, for example, is ranked high in this direction, nearer to the octalaminar calcarine sulci, among the thinnest areas of the cortex, than to other periarchicortical areas. This can be explained by the fact that the pericallosal sulcus also has heterotypic granular isocortical portions (Triarhou, 2007). It is also thinner than most cortices, as can be seen in Fig. 3. We cannot, however, dismiss that other mechanisms possibly contribute to the scores in this direction.

Another identification can be made towards myelination. Mesocortical regions are generally less myelinated than neocortex. Since the neocortex is more prevalent, the association between thicker cortex and atrophy is more apparent, and for this reason the model attributes a higher rate of expected atrophy to regions containing mesocortex, for example the temporal poles and the parahippocampal gyri. Their low atrophy could then be explained by their low myelin content. A model that includes measures of myelination thickness or content (Rowley et al., 2015; Westlye et al., 2009) could elucidate this question. The case of the temporal poles is complicated even more by their almost heterotypic nature, with sparsely populated internal and external granular layers (Triarhou, 2007), a characteristic shared with thick agranular cortices. Thus, their thickness and borderline agranular characteristics are not key factors for the observed atrophy rates.

Valk et al. (2020) reports similar results, with anterior-posterior and inferior-superior gradients from the decomposition of the structural covariance of cortical thickness. Interestingly, in their work authors remove the effect of age on the cortical thickness covariance. In our work, we instead study the cortical trajectories across the adult lifespan.

Sulci have on average thinner infragranular layers than gyri. See Shamir et al. (2019), Welker (WelkerJ.E.G., 1990), Wagstyl et al. (Wagstyl et al., 2158), Hilgetag and Barbas (2006). Their trajectories are more similar to correspondingly thicker gyri, due to similar supragranular content. In fact, among sulci, a significant superlinear association can be detected. See Figure S5. This means that thicker sulci display higher rates of atrophy than thinner sulci. Additionally, the rate of atrophy of thicker sulci is proportionately higher. Given these major morphological differences between gyri and sulci, we argue that our results might better reflect gyral features. On the other hand, taking into account these differences may allow for a unified undertanding of the association between thickness and thinning.

To the best of authors’ knowledge, our work is the first to demonstrate quantitatively a relationship between laminar architecture and cortical thinning.

Our study, as many others in the area of human brain mapping, is at risk of hidden variable bias. Sociodemographic factors, which have some correlation with sex and age, are unaccounted for, and these have some bearing on neuroimaging findings. Other possible confounders include in-scanner motion, since elders are more likely to move in the scanner and this has been shown to bias cortical thickness estimates (Savalia et al., 2017).

A longitudinal study would be able to better estimate the rates of change in morphometry, using the difference between subsequent estimates. Longitudinal studies are costlier and more complex though and since thinning is a linear process, our cross-sectional paradigm should result in reasonably accurate estimates. That design does not improve the estimation of the “initial” thickness either compared to the cross-sectional design. A longitudinal study could further elucidate the relationship between the initial and the change in morphometry estimates in a population. However, it would not better answer our specific question on how the morphometry at 18 years of age relates to the subsequent yearly atrophy.

A possible caveat in our morphometric analysis, is the non-linear behavior of age-related phenomena in several brain structures, as reported in the literature (Scahill et al., 2003; Goodro et al., 2012; Storsve et al., 2014; Walhovd et al., 2011; Fjell et al., 2009a, 2014b). At the degrees of freedom afforded by the large sample size, we believe the linear models sufficiently capture the direction of effects, even though rates of change might be off at different ages. Moreover, using data from 17075 healthy subjects, Frangou et al. (2021) demonstrates that, starting at the second decade trajectories are approximately linear in most cortices up to the eighth decade of life. As a byproduct, the linear modeling also facilitates the analysis, since we retrieve a single constant atrophy rate that can be compared with the estimate of the thickness at the start of the trajectory. Non-linear trajectories, on the other hand, present varying rates of atrophy.

Another possible caveat of our analyses is that thickness in thinner cortices is innately harder to estimate and more susceptible to noise and artifacts. Different reliability estimates for thinner cortices (Kharabian Masouleh et al., 2020) do not contribute substantially to the effects in Fig. 2(a) and (b). See Supplementary Material S-VI for a similar analsis with additional weighting based on effect sizes. This is due to the robust modeling, that attenuates the leverage of deviations in data points. In particular, problematic paralimbic regions contribute minimally to the model. See Figure S7(c).

The use of anatomical regions, albeit justified to allow direct comparisons to previous literature, does not necessarily respect cytoarchitectonic boundaries. This is specially glaring in the definition of the superior frontal gyrus, which encompasses homotypic and heterotypic portions (Triarhou, 2007), and also the previously mentioned pericallosal sulcus. Future work will explore the difference in atlases and also a vertexwise analysis. This could ideally disambiguate the effects in different areas of the cortex.

Since the NKI-RS has an adequate sample size, we opted to exclude left-handed subjects from the sample due to the possibility of confounding. However, this subpopulation can also be studied in future studies to elucidate if handedness plays a role in the associations presented in our study.

Studies on the laminar thickness could further elucidate the role of neuronal populations in age-related cortical thickness. New cohorts using ultra-high field MRI scanners will prove fundamental in this issue.

Our results reassert gray-matter atrophy in the whole brain, affecting cortical thickness and surface area. In particular, we show for the first time how the cortical age-related thinning profiles have some association to the starting cortical thickness, with thicker cortex displaying, proportionally, smaller rates of thinning. We propose a relationship between initial cortical thickness and rate of thinning is associated with cortical laminar architecture. Under this light, pure neocortex presents higher rates of atrophy, higher still in agranular than in granular neocortex, than mixed cortices. However, proportionately, thicker cortices lose smaller proportions of thickness compared with thinner cortices.

## Declaration of competing interest

The authors declare that they have no known competing financial interests or personal relationships that could have appeared to influence the work reported in this paper.

## Disclosure statement

Authors declare that there is no conflict of interest.
