# Supplementary material for: Sublinear association between cortical thickness at the onset of the adult lifespan and age-related annual atrophy parallels spatial patterns of laminar organization in the adult cerebral cortex
Source: Neuroimage Rep. 2021 May 17;1(2):100011. doi: 10.1016/j.ynirp.2021.100011 (PMC12172795; doi:10.1016/j.ynirp.2021.100011)
Supplement: Multimedia component 1 [file mmc1.pdf]

Supplementary Material: Sublinear association between cortical thickness at the onset of the adult lifespan and age-related annual atrophy parallels spatial patterns of laminar organization in the adult cerebral cortex

S-I. Comparison based on granule populations

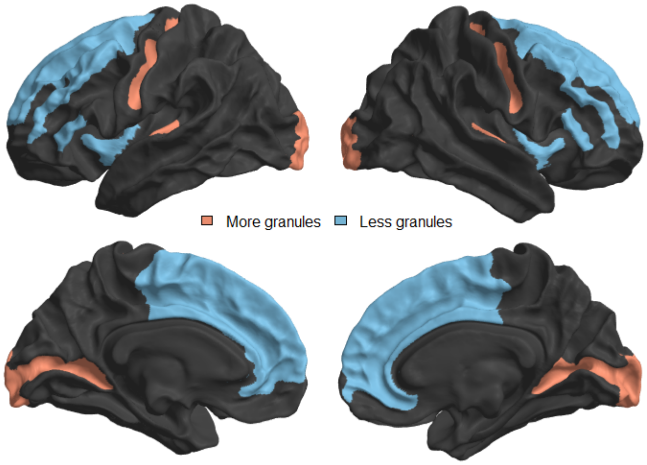

Supplementary Figure S1: Regions selected as containing more granule cells are shown in red, while regions selected as containing less granule cells are shown in blue. Red: central and calcarine sulci, occipital poles and anterior transverse temporal gyri. Blue: superior, middle, inferior pars triangularis frontal gyri, the superior frontal sulci, the short insular gyri, the anterior, middle-anterior and middle-posterior cingulate gyri and sulci. This selection is based on von Economo [21] and Shamir et al. [45].

S-II. Effect of age on cortical thickness

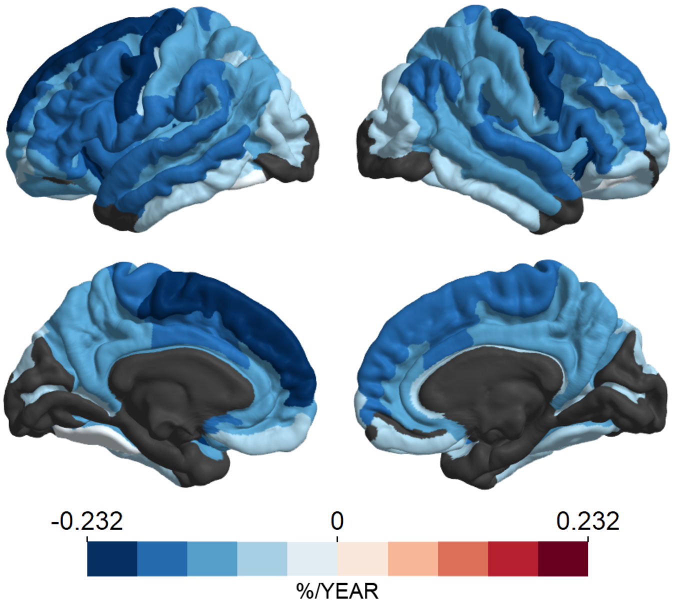

Supplementary Figure S2: Yearly percentage change of the expected value of cortical thickness at a reference age of 18 years, modelled by Equation 1. Only significant effects at a 0.05 FDR significance level are colored.

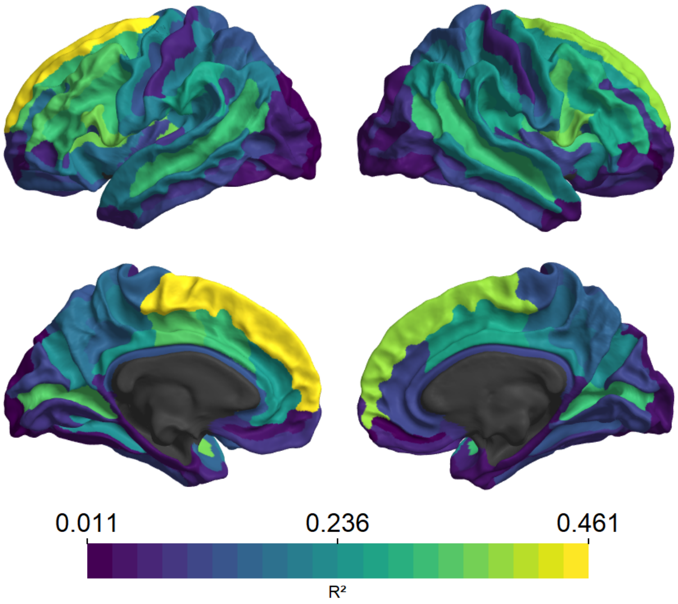

Supplementary Figure S3: Inter-individual variability as measured by the R^2 obtained by a linear model as described in Equation 1.

S-III. Effect of age on cortical surface area

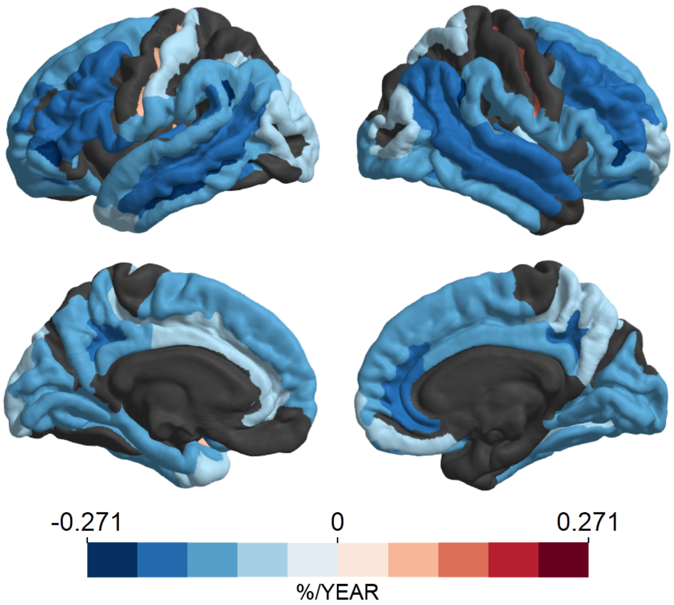

Supplementary Figure S4: Yearly percentage change of the expected value of cortical surface area at a reference age of 18 years, modelled by Equation 1 Only significant effects at a 0.05 FDR significance level are colored.

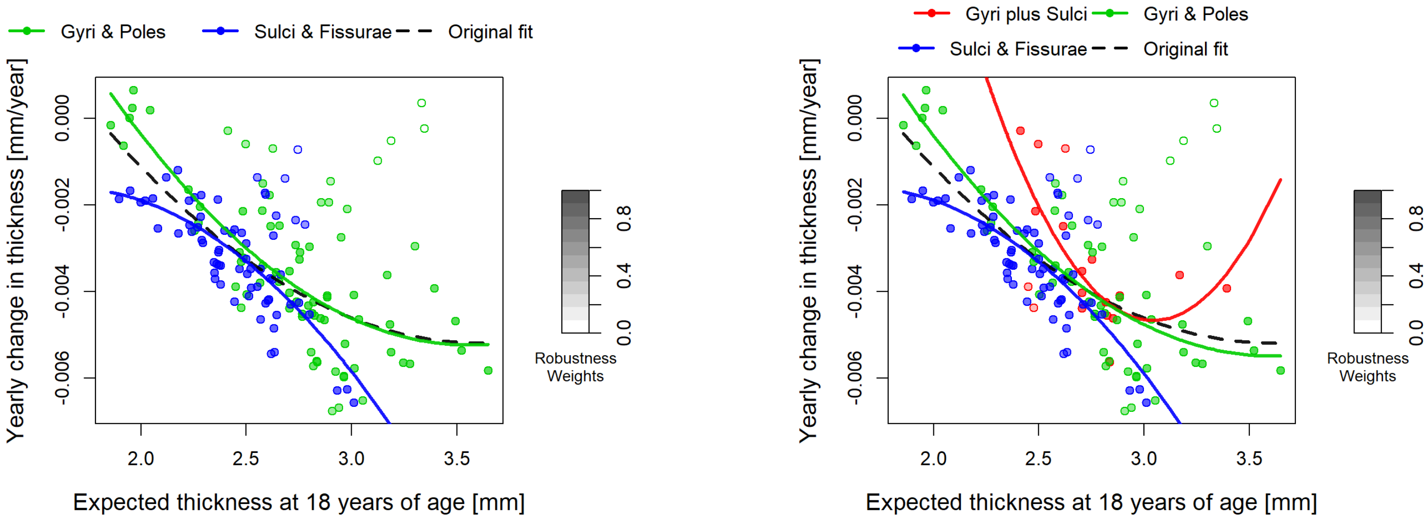

(a) (b)

Supplementary Figure S5: Yearly thickness change versus the expected thickness at the age of 18 years Modelling of gyral-sulcal differences in the dependence between yearly change and initial thickness of 148 cortical regions defined in the Destrieux atlas. (a) taking into account gyral and sulcal differences, where "gyrus and sulcus" regions were grouped with gyri; (b) additionally modelling "gyrus and sulcus" regions as both sulcal and gyral. The black dashed line represents the quadratic fit shown in Figure 1(d). Opacity represents robust weighting.

S-V. Distance from archicortex and paleocortex

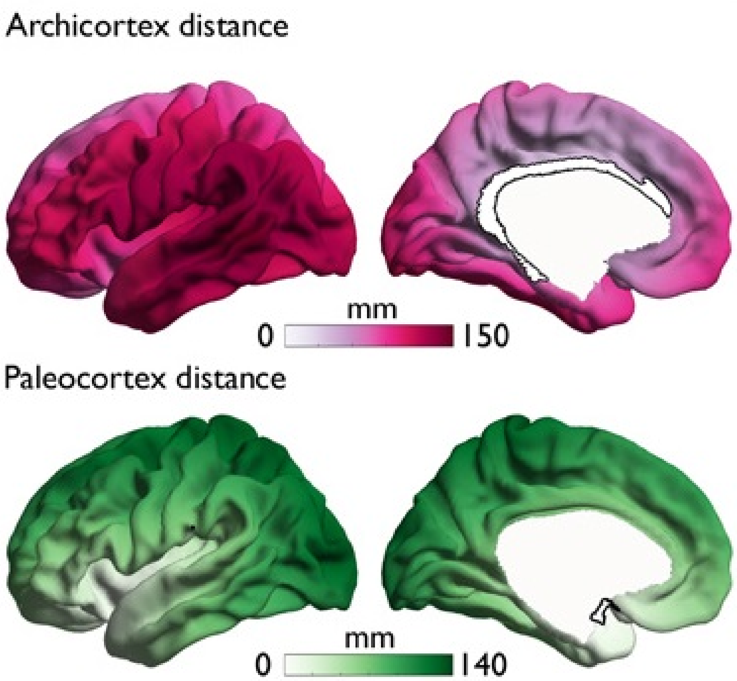

Supplementary Figure S6: Distance from archicortex and paleocortex in humans. This figure is an adaptation of Valk et al. [18, Figure 4], and is used under a CC BY 4.0 license. See `https://creativecommons.org/licenses/by/4.0/`.

S-VI. Weighting for effect size

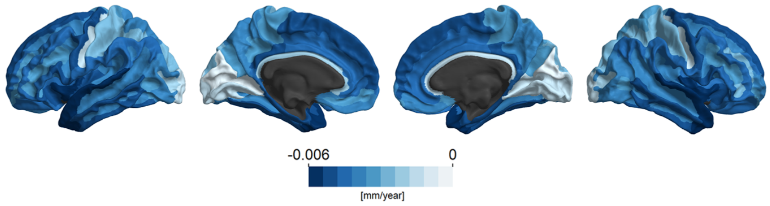

(a)

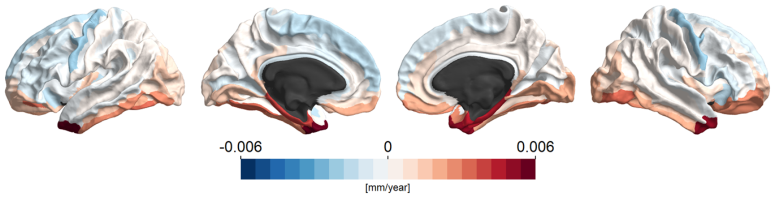

(b)

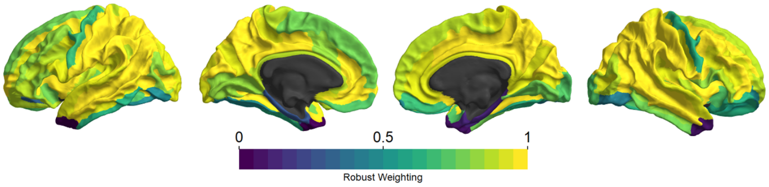

(c)

Supplementary Figure S7: Expected yearly rate of atrophy (a) and residuals (b) according to the model in Equation 3 when further weighing observations by their effect size. Results are directly comparable to Figure 2. This is due to the robust weighting (c) from the model fitting in Figure 1(d). Some regions do not contribute substantially if at all to the model.
